# Effects of early phase 1 cardiac rehabilitation on cardiac function evaluated by impedance cardiography in patients with coronary heart disease and acute heart failure

**DOI:** 10.3389/fcvm.2022.958895

**Published:** 2022-08-24

**Authors:** Yishu Wang, Yanchao Xiao, Jianjun Tang, Yutao Liu, Hui Li, Zengjin Peng, Danyan Xu, Li Shen

**Affiliations:** ^1^Department of Internal Cardiovascular Medicine, Second Xiangya Hospital, Central South University, Changsha, China; ^2^The First People’s Hospital of Xiangtan City, Xiangtan, China

**Keywords:** phase 1 cardiac rehabilitation, impedance cardiography, acute heart failure, cardiac function, hemodynamics

## Abstract

**Purpose:**

The purpose of the study was to access the impact of phase 1 cardiac rehabilitation (CR) on cardiac function and hemodynamic changes in patients with coronary heart disease (CHD) and acute heart failure (AHF).

**Materials and methods:**

A total of 98 patients with CHD and AHF were recruited and randomized into two groups. Control group received standard pharmacotherapy and CR group received standard pharmacotherapy combined phase 1 CR. NT-proBNP and hemodynamic parameters measured by impedance cardiography (ICG) were estimated at baseline and at the end of treatment period.

**Results:**

Phase 1 CR combined routine medical treatment could lower NT-proBNP levels. The percentage of high-risk patients was significantly decreased in CR group, although the post-treatment NT-proBNP level between control group and CR group showed no significant differences. Similarly, most hemodynamic parameters improved in the CR group, but not in the control group, suggesting that phase 1 CR in combination with the standard pharmacotherapy improved hemodynamic characteristics by elevating cardiac output, ameliorating preload, improving systolic and diastolic function, and relieving afterload, although the post-treatment hemodynamic parameters showed no statistically significant differences between the control group and the CR group.

**Conclusion:**

Phase 1 CR combined routine medication can improve cardiac function and hemodynamic characteristics in patients with CHD and AHF. Thus, recommendation of phase 1 CR to stable patients is necessary.

## Introduction

Coronary heart disease (CHD) is one of the most common causes of heart failure (HF). Despite the improvement in long-term prognosis of patients with HF due to the development of pharmacotherapy, intervention, implantable cardioverter defibrillator, and cardiac resynchronization therapy, the mortality and re-admission rate of patients with HF, however, remain high. Thus, improving prognosis and outcomes of patients with HF is of high priority.

Cardiac rehabilitation (CR), an accessible and economical therapy, has attracted progressive attention in the recent years. European Society Of Cardiology (ESC) and American Heart Association/American College of Cardiology (AHA/ACC) have recognized CR as a class I recommendation for patients with HF (1). CR includes three phases: inpatient CR (phase 1 CR), early stage of outpatient CR (phase 2 CR), and long-term community-based CR (phase 3 CR). Phase 1 CR (or inpatient CR) provides hospitalized patients with cardiac rehabilitation and preventive measures including exercise training, patient education, and behavior interventions. Several guidelines and expert consensus recommend phase 1 CR to hemodynamical stable patients with acute heart failure (AHF) and patients with HF recurrence (2–5).

It has been demonstrated that phase 1 CR contributed to alleviate symptoms (6), improve functional capacity and activity of daily living (7–9), shorten hospital stay length (10, 11), and reduce re-admission rate (11, 12) and all-cause mortality (11, 13). Specifically, patients receiving phase 1 CR had a 26% increase in 6-min walk test (6MWT) compared with controls (14), suggesting an enhancement in cardiopulmonary function and exercise capacity. Consistently, early movement within 48 h improved oxygen uptake efficiency slope in patients with acute myocardial infarction (AMI) (15). Besides, early movement training also ameliorated the inflammatory level in patients with AMI (16).

However, the prevalence of phase 1 CR remains low. A cross-sectional investigation including 454 hospitals revealed that only 24% hospitals provide phase 1 CR program (17). Meanwhile, the awareness of phase 1 CR in patients remains relatively insufficient (18). Therefore, there is a need to promote phase 1 CR.

The continuous monitoring and evaluation of patients with HF in phase 1 CR is critical, but an accurate and efficient method lacks. Impedance cardiography (ICG), a non-invasive approach of constant monitoring instantaneous changes in thoracic electrical impedance based on the Ohm’s law, can provide reliable hemodynamic values and has been used to estimate cardiac function in patients with HF (19). Previous studies have confirmed the accuracy of ICG by comparing it to echocardiography (20). Meanwhile, as the change of hemodynamics status happens prior to occurrence of symptoms, the feature that ICG can capture small hemodynamic changes and thus can identify asymptomatic abnormalities makes ICG a more sensitive method than echocardiography. Most importantly, the intensity and duration of phase 1 CR can be adjusted promptly based on patients’ condition reflected by ICG-measured hemodynamic changes. Furthermore, the prospective evaluation and identification of cardiac decompensation by ICG test (PREDICT) study finds that the combination of parameters measured by ICG can predict the short-term mortality and re-admission rate of patients with HF (21). Thus, ICG can be applicated as a useful approach in evaluating the effectiveness of phase 1 CR.

However, the effects of phase 1 CR on hemodynamic changes in patients with AHF remain unknown. In this study, we attempt to explore the impact of phase 1 CR on cardiac function and hemodynamics in patients with CHD and AHF through the pre- and post-treatment hemodynamic changes detected by ICG.

## Materials and methods

### Study sample

This study was approved by the Clinical Research Ethics Committee, the Second Xiangya Hospital of Central South University, China. All participants provided informed consent.

This study was a randomized controlled trial. A total of 106 patients with CHD and AHF who were admitted for treatment in cardiac care units from 2019 to 2020 were recruited and randomly assigned to one of two treatment groups, the control group or the CR group.

Randomization and allocation sequence was based on a block size fixed to 2 and generated through a computerized random number generator by a staff not involved in the trial. The patients in control group were treated with ordinary standard medical treatment. The patients in CR group received medical treatment plus 1-week phase 1 CR program during hospitalization.

The inclusion criteria were as follows: (1) age > 18 years; (2) the percutaneous transluminal coronary intervention revealed > 75% narrowing of the proximal anterior descending artery or three main coronary arteries; (3) the echocardiography showed enlarged heart with the diagnosis meets the criteria of left ventricular end diastolic diameter (LVEDd) > 5 cm; (4) the left ventricular ejection faction (LVEF) between 30 and 50%; (5) apparent clinical signs and symptoms of AHF appeared; and (6) laboratory test showed elevated NT-proBNP level.

The exclusion criteria were as follows: (1) any life-threatening comorbidities, (2) patients with unstable hemodynamics; (3) acute phase of pulmonary diseases, including asthma attacks, pulmonary embolism, pneumothorax, and impaired cognition, (4) severe infections, such as infectious endocarditis and septicemia; (5) uncontrolled arrhythmia; (6) severe valvular disease; (7) trauma or surgical history in the past 6 months; (8) aortic dissection; (9) cancer; (10) cognitive limitation; and (11) refuse to provide consent. Patients with the main diagnosis other than CHD and AHF were also excluded.

### Phase 1 cardiac rehabilitation program

Phase 1 CR was performed under the instruction and observation of experienced physicians and adjusted according to the patients’ conditions. The specific procedure was followed by the fourth edition of guidelines for CR and secondary prevention program (22).

Phase 1 CR began when patients fitted those following conditions: (1) no chest pain in the past 8 h; (2) no evident symptoms or signs of decompensated heart failures; (3) no new onset arrhythmia nor changes on electrocardiograph (ECG); and (4) no elevation of NT-proBNP.

The phase 1 CR program lasted for 7 days. On each day, the duration is 30 min, including 10-min warm-up activity, 10-min aerobic exercise, and 10-min Meridians patting or flexibility training according to the patients’ conditions.

The evaluation of the phase 1 CR program is the combination of targeted heart rate, which is to raise heart rate to 20 ± 5 beat per minute above the rest heart rate, and Borg scale, a rating of perceived exertion scale.

The criteria of termination of phase 1 CR were as follows: (1) chest pain, palpitations, dyspnea, sweating, and other obvious discomfort symptoms; (2) ECG showed frequent ventricular tachycardia, atrial tachycardia, atrial fibrillation, and other malignant arrhythmias; (3) systolic blood pressure did not increase but decreased by 10 mmHg or more, or systolic blood pressure elevated by 180 mmHg; and (4) the patient requested to stop.

### Data collection

The echocardiography was performed by experienced physician within 24 h of admission.

The hemodynamic parameters were detected by experienced staff using ICG (CSM3000, the Cheer Sails Medical). ICG was performed after 5 min of rest in the supine position. The electrodes were placed following the instructions. The hemodynamic variables included cardiac output (CO), cardiac index (CI), stroke volume (SV), stroke index (SI), thoracic fluid content (TFC), pre-ejection period (PEP), left ventricular ejection time (LVET), systolic time ratio (STR), systemic vascular resistance (SVR), stroke systemic vascular resistance (SSVR), and stroke systemic vascular resistance index (SSVRI). The venous blood was obtained for detection of NT-proBNP followed by ICG measurements.

### Statistics

The data were analyzed by SPSS version 20.0. Measurement data followed normal distribution were expressed as mean ± standard deviation, whereas non-normal distributional data were expressed as median (interquartile range). Enumeration data were expressed as proportion and evaluated by Chi-square test. Paired *t*-test was used to compare the changes in normal distributional parameters, and Kruskal–Wallis test was used to compare the changes in non-normal distributional parameters. A repeated measures ANOVA was used to compare the pre- and post-treatment changes in control and CR group. Spearman’s correlation analysis was used to evaluate the correlation between the parameters determined by ICG and blood NT-proBNP. A value of *p* < 0.05 was considered statistically significant.

## Results

### Basic characteristics

**FIGURE 1:**
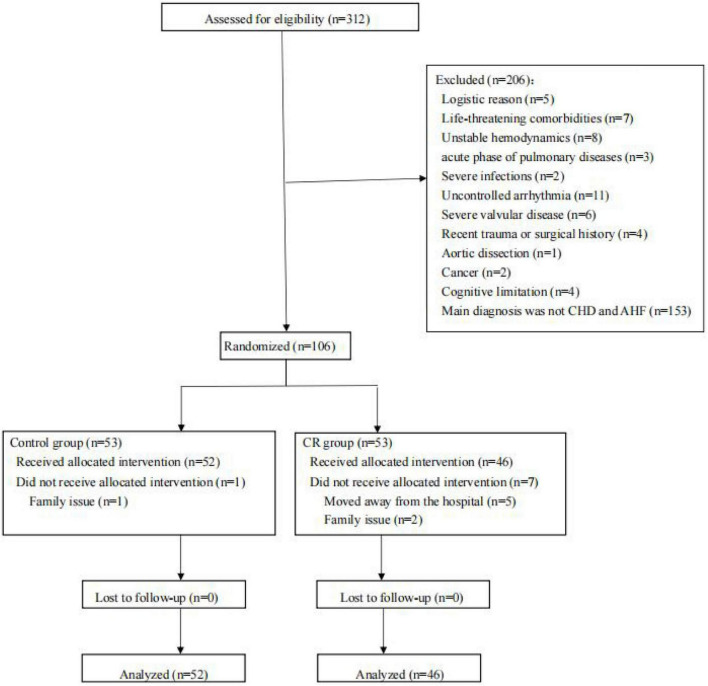
Flow diagram of patients.

The study included 106 patients (average age 66.96 ± 2.76 years old, 72.4% male). The control group contained 53 patients, 52 of whom finished the treatment. The CR group contained 53 patients, 46 of whom were analyzed (Figure 1). The fundamental data are shown in Table 1. There were no differences in height, weight, body mass index (BMI), blood pressure and rest heart rate, companion diseases, and medical history between control and CR group before treatment. The echocardiography showed comparable in cardiac structure and cardiac function between two groups (Table 2).

**TABLE 1:** Comparison of baseline clinical characteristics of patients.

Parameters (unit)	All patients (*n* = 98)	Control group (*n* = 52)	CR group (*n* = 46)	*P*-value
Age (years)	66.96 ± 2.76	65.26 ± 2.80	68.38 ± 2.73	0.990
Gender [male, *n* (%)]	71 (72.4%)	39 (75.0%)	32 (69.6%)	0.548
Height (cm)	164.22 ± 1.27	162.35 ± 1.43	165.77 ± 1.14	0.166
Weight (kg)	67.92 ± 2.64	65.30 ± 2.84	70.12 ± 2.48	0.703
BMI (kg/m^2^)	24.20 ± 0.82	23.79 ± 0.89	24.55 ± 0.76	0.747
Rest HR (beat/minute)	68.29 ± 2.00	66.83 ± 2.38	69.50 ± 1.69	0.892
SBP (mmHg)	122.01 ± 3.11	120.78 ± 3.30	123.04 ± 2.96	0.340
DBP (mmHg)	69.97 ± 1.82	65.04 ± 2.01	74.08 ± 1.67	0.778
MAP (mmHg)	84.59 ± 1.76	81.04 ± 1.83	87.54 ± 1.70	0.710
**Accompany disease, *n* (%)**				
Hypertension	69 (70.4%)	36 (69.2%)	33 (71.7%)	0.827
Diabetes	72 (73.5%)	41 (78.9%)	31 (67.4%)	0.253
**Medication, *n* (%)**				
Furosemide	77 (78.6%)	42 (80.8%)	35 (76.1%)	0.573
Loop diuretics	80 (81.6%)	44 (84.6%)	36 (78.3%)	0.445
Aspirin	98 (100.0%)	52 (100.0%)	46 (100.0%)	1.000
Clopidogrel	98 (100.0%)	52 (100.0%)	46 (100.0%)	1.000
Statins	98 (100.0%)	52 (100.0%)	46 (100.0%)	1.000
Beta blocker	82 (83.7%)	44 (84.6%)	38 (82.6%)	0.789
ACEI/ARB/ARNI	88 (89.8%)	47 (90.4%)	41 (89.1%)	0.838
Digoxin	41 (41.8%)	21 (40.4%)	20 (43.5%)	0.757

Data are expressed as mean ± standard, or percentages. n, number; CR, cardiac rehabilitation; BMI, body mass index; rest HR, heart rate at rest; SBP, systolic blood pressure; DBP, diastolic blood pressure; MAP, mean arterial pressure; ACEI, angiotensin-converting enzyme inhibitors; ARB, angiotensin receptor blocker; ARNI, angiotensin receptor neprilysin inhibitors.

**TABLE 2:** Comparison of baseline parameters of echocardiography between control group and CR group.

Parameters (unit)	control group (*n* = 52)	CR group (*n* = 46)	*P*-value
LAD (mm)	34.09 ± 1.15	32.31 ± 0.84	0.142
LVED (mm)	59.87 ± 0.89	61.00 ± 1.27	0.580
IVST (mm)	9.91 ± 0.32	9.81 ± 0.14	0.062
LVPWT (mm)	9.57 ± 0.25	9.42 ± 0.16	0.254
LVEF (%)	35.73 ± 16	36.13 ± 10	0.745

Data are expressed as mean ± standard. LAD, left atrial diameter; LVED, left ventricular end-diastolic diameter; IVST, interventricular septal thickness; LVPWT, left ventricular posterior wall thickness; LVEF, left ventricular ejection fraction.

### T-proBNP levels in pre- and post-phase 1 cardiac rehabilitation

As shown in Table 3, there were no differences in NT-proBNP level between control and CR group before treatment.

**TABLE 3:** Comparison of NT-proBNP of patients with CHD and AHF before and after the treatment.

Parameter (unit)	Control group (*n* = 52)			CR group (*n* = 46)			*P* *	*P* ^#^
		
	Pre-treatment	Post-treatment	*p*	Pre-treatment	Post-treatment	*p*		
NT-proBNP (pg/ml)	1913.62 (926.33; 4378.22)	1439.61 (283.7275; 2594.87)	0.004	2643.00 (1527.59; 4360.00)	1889.00 (704.85; 3315.00)	0.000	0.536	0.188

Data are expressed as median (interquartile range). CR, cardiac rehabilitation; NT-proBNP, N-terminal pro brain natriuretic peptide.

*p*: values of comparison between changes of pre-treatment and post-treatment observed in CR group or control group.

*p: values of comparison between baseline parameter in CR group versus those in control group.

^#^*p*: values of comparison between parameter after treatment observed in CR group versus those in control group.

*p* < 0.05 was considered statistically significant.

In control group, the NT-proBNP level decreased from 1913.62 (926.33; 4,378.22) pg/ml to 1,439.61 (283.7275; 2,594.87) pg/ml after the treatment (*p* < 0.05). In CR group, the NT-proBNP level decreased from 2,643.00 (1,527.59; 4,360.00) pg/ml to 1,889.00 (704.85; 3,315.00) pg/ml after the treatment (*p* < 0.05).

After treatment, the NT-proBNP level in CR group was non-significantly lower than that of control group.

The level of NT-proBNP was found to correlated with the short-term prognosis of patients with AHF and that patients with NT-proBNP higher than 5,180 pg/ml had higher risk of sudden death (23). Thus, the subgroup analyses defined patients with NT-proBNP > 5,180 pg/ml as high NT-proBNP group, whereas NT-proBNP ≤ 5,180 pg/ml as low NT-proBNP group. Before the treatment, 22.4% of patients (control group 19.2%, CR group 26.1%) were divided into high NT-proBNP group, while after the treatment, 8.2% of patients (control group 13.5%, CR group 2.2%) have NT-proBNP higher than 5,180 pg/ml. In control group, the patients with high NT-proBNP group decreased by 5.7%, whereas in CR group, that percentage decreased by 23.4%. In addition, the number and the percentages of patients with NT-proBNP higher than 5,180 pg/ml in the CR group significantly decreased (*p* < 0.05), which suggested that phase 1 CR could further lower plasma NT-proBNP level and improve short-term prognosis of patients with CHD and AHF based on the routine medicine.

### Impedance cardiography parameters comparison pre- and post-phase 1 cardiac rehabilitation

#### Cardiac output parameters

Table 4 showed the following cardiac output parameters: cardiac output (CO), cardiac output index (CI), stroke volume (SV), and stroke volume index (SI) of control group and CR group. As it showed, there were no differences between two groups before treatment. After the treatment, no significant changes were found between two groups.

**TABLE 4:** Comparisons of parameters of cardiac output measured by impedance cardiography of patients with CHD and AHF before and after the treatment.

Parameters (unit)	Control group (*n* = 52)				CR group (*n* = 46)				*P* *	*P* ^#^
		
	Pre-treatment	Post-treatment	Changes	P	Pre-treatment	Post-treatment	Changes	*P*		
CO (L/min)	4.05 (3.72;4.87)	4.70 (3.70;5.10)	0.40 (–0.18;0.80)	0.204	3.90 (3.55;4.80)	4.70 (4.05;5.05)	0.20 (0.05;0.95)	0.005	0.775	0.801
CI (L/min/m^2)^	2.65 (2.07;2.80)	2.75 (2.22;3.17)	0.20 (–0.75;0.58)	0.184	2.30 (2.05;2.65)	2.70 (2.35;2.85)	0.20 (0.00;0.55)	0.008	0303	0.688
SV (mL)	57.50 (47.00;69.50)	61.10 (50.25;79.00)	5.00 (–10.25;16.25)	0.414	54.00 (42.50;70.00)	68.00 (54.50;75.50)	5.00 (–2.00;18.50)	0.03	0.623	0.698
SI (mL/m^2^)	36.50 (25.75;44.50)	36.00 (30.50;47.75)	2.50 (–5.75;10.00)	0.408	30.00 (25.75;36.50)	35.50 (29.50;43.00)	1.50 (–0.33,9.00)	0.057	0.214	0.473

Data are expressed as median (interquartile range). CR, cardiac rehabilitation; CO, cardiac output; CI, cardiac index; SV, stroke volume; SI, stroke index.

*p*: values of comparison between changes of pre-treatment and post-treatment observed in CR group or control group.

*p: values of comparison between baseline parameter in CR group versus those in control group.

^#^*p*: values of comparison between parameter after treatment observed in CR group versus those in control group.

*p* < 0.05 was considered statistically significant.

In control group, the cardiac output parameters showed no changes before and after the treatment, whereas in CR group, CO, CI, and SV increased after the treatment (*p* < 0.05). It indicated that routine treatment had limitations in improving cardiac output in short term, while medical treatment combined phase 1 CR could improve cardiac output in patients with CHD and AHF.

#### Afterload parameter

As Table 5 showed, there were no differences in TFC between two groups before and after the treatment. After the treatment, the TFC of control group decreased from 0.034 (0.029; 0.036)/Ω to 0.030 (0.025; 0.035)/Ω (*p* > 0.05), at the same time, the TFC of CR group decreased from 0.035 (0.029;0.041)/Ω to 0.031 (0.029;0.035)/Ω (*p* > 0.05). However, the statistics showed no significant changes in TFC before and after treatment in both groups.

**TABLE 5:** Comparison of preload parameter measured by impedance cardiography of patients with CHD and AHF before and after the treatment.

Parameter (unit)	Control group (*n* = 52)				CR group (*n* = 46)				*P* *	*P* ^#^
		
	Pre-treatment	Post-treatment	Change	*P*	Pre-treatment	Post-treatment	Change	*P*		
TFC (1/Ω)	0.034 (0.029;0.036)	0.030 (0.025;0.035)	–0.002 (–0.005;0.001)	0.141	0.035 (0.029;0.041)	0.031 (0.029;0.035)	–0.001 (–0.008;0.004)	0.100	0.205	0.234

Data are expressed as median (interquartile range). CR, cardiac rehabilitation; TFC, Thoracic Fluid Content.

*p*: values of comparison between changes of pre-treatment and post-treatment observed in CR group or control group.

*p: values of comparison between baseline parameter in CR group versus those in control group.

^#^*p*: values of comparison between parameter after treatment observed in CR group versus those in control group.

*p* < 0.05 was considered statistically significant.

To further determine whether phase 1 CR has influence on TFC level, we divided patients into two subgroups: patients with TFC ≥ 0.035/Ω were defined as high-preload group, whereas patients with TFC < 0.035/Ω were defined as low-preload group. Before the treatment, 50.0% of patients (50.0% in control group, 50.0% in CR group) were in high-preload subgroup. After the treatment, 29.6% of patients (30.8% in control group, 28.3% in CR group) had TFC higher than 0.035/Ω. The percentage of patients with high preload was decreased in both control group and CR group.

To further identify the effects of phase 1 CR under same preload situation, we compared TFC changes of high- or low-preload settings in control group and CR group. The results are shown in Table 6. In control group, TFC changes were not statistically significant in either high- or low-preload settings. In CR group, though no significant change was found in low-preload subgroup, the TFC level decreased from 0.0395 (0.0365; 0.0427)/Ω to 0.0315 (0.0290; 0.0350)/Ω (*p* < 0.05) in high-preload setting. Taken together, these findings indicated that phase 1 CR plus routine treatment decrease TFC in high-preload patients.

**TABLE 6:** Comparison of preload of patients with CHD and AHF before and after the treatment under same preload setting.

TFC (/Ω)	Control group (*n* = 52)		CR group (*n* = 46)	
		
	High-preload subgroup (TFC ≥ 0.035/Ω) (*n* = 26)	Low-preload subgroup (TFC < 0.035/Ω) (*n* = 26)	High-preload subgroup (TFC ≥ 0.035/Ω) (*n* = 20)	Low-preload subgroup (TFC < 0.035/Ω) (*n* = 26)
Pre-treatment	0.0360 (0.0350;0.0400)	0.0290 (0.0250;0.0310)	0.0395 (0.0365;0.0427)	0.0290 (0.0260;0.0310)
Post-treatment	0.0320 (0.0258;0.0408)	0.0280 (0.0245;0.0325)	0.0315 (0.0290;0.0350)	0.0305 (0.0290;0.0330)
*P*	0.161	0.720	0.010	0.155

Data are expressed as median (interquartile range). CR, cardiac rehabilitation; TFC, Thoracic Fluid Content.

*p*-values of comparison between TFC changes of pre-treatment and post-treatment observed in high or low-preload settings in control group or CR group.

*p* < 0.05 was considered statistically significant.

#### Contraction parameters

As Table 7 showed, there were no differences in PEP, LVET, and STR between two groups before and after the treatment.

**TABLE 7:** Comparisons of cardiac function of contraction measured by impedance cardiography of patients with CHD and AHF before and after the treatment.

Parameters (unit)	Control group (*n* = 52)				CR group (*n* = 46)				*P* *	*P* ^#^
		
	Pre-treatment	Post-treatment	Changes	*P*	Pre-treatment	Post-treatment	Changes	*P*		
PEP (ms)	101.00 (90.50;117.00)	97.00 (86.50;113.50)	–4.00 (–20.00;11.50)	0.387	114.00 (109.00;133.00)	102.00 (87.00;115.00)	–12.00 (–23.00;-3.00)	0.001	0.057	0.473
LVET (ms)	270.00 (257.00;306.00)	280.00 (259.00;313.50)	7.00 (–8.50;40.25)	0.227	248.00 (242.00;289.00)	266.00 (242.00;296.00)	12.00 (–17.00;34,00)	0.140	0.139	0.247
STR (–)	0.40 (0.30;0.40)	0.30 (0.30;0.40)	0.00 (–0.10;0.00)	0.178	0.50 (0.35;0.50)	0.40 (0.30;0.50)	–0.10 (–0.10;0.00)	0.006	0.067	0.158

Data are expressed as median (interquartile range). CR, cardiac rehabilitation; PEP, pre-ejection period; LVET, left ventricular ejection time; STR, systolic time ratio.

*p*: values of comparison between changes of pre-treatment and post-treatment observed in CR group or control group.

*p: values of comparison between baseline parameter in CR group versus those in control group.

^#^*p*: values of comparison between parameter after treatment observed in CR group versus those in control group.

*p* < 0.05 was considered statistically significant.

Medical treatment decreased the PEP in control group from 101.00 (90.50; 117.00) to 97.00 (86.50; 113.50) ms (*p* > 0.05), whereas phase 1 CR plus medication shorter PEP from 114.00 (109.00; 133.00) to 102.00 (87.00;115.00) ms (*p* < 0.05).

Pre- and post-treatment LVET showed no differences in both control and CR groups.

The STR in control group was shorter from 0.40 (0.30; 0.40) to 0.30 (0.30; 0.40) (*p* > 0.05), whereas in CR group, the STR was changed from 0.50 (0.35; 0.50) to 0.40 (0.30; 0.50) (*p* < 0.05). The results suggested that routine treatment plus phase 1 CR shorter PEP and STR, thus improving constriction function of left ventricular.

Moreover, to identify the effects of phase 1 CR in improving left ventricular constriction, subgroup analysis was made. STR > 0.4 was defined as dysfunctional constriction. Before the treatment, 39.8% of patients (control group 21.2%, CR group 60.8%) were defined as dysfunctional constriction, while after the treatment, 16.3% of patients (control group 5.8%, CR group 28.3%) had dysfunctional constriction. The standardized percentage of patients with constriction problem decreased 15.1 and 32.0%, respectively, in control group and CR group, which implied that phase 1 CR can improve constriction function of left ventricular.

#### Pressure load parameters

As Table 8 showed, there were no differences in SVR, SSVR, and SSVRI between two groups before and after the treatment.

**TABLE 8:** Comparison of afterload parameters measured by impedance cardiography of patients with CHD and AHF before and after the treatment.

Parameters (unit)	Control group (n = 52)				CR group (n = 46)				*P* *	*P* ^#^
		
	Pre-treatment	Post-treatment	Changes	P	Pre-treatment	Post-treatment	Changes	*P*		
SSVR (dynes/cm^5^/beat)	244.05 (213.77;352.90)	236.30 (207.37;278.77)	–21.65 (–69.58;42.73)	0.313	300.70 (210.40;352.07)	251.45 (175.35;318.85)	–42.45 (–61.88; –6.60)	**0.016**	0.413	1.000
SSVRI (dynes/cm^5^/m^2^/beat)	155.00 (129.12;203.30)	141.35 (129.80;159.80)	–16.05 (–52.13;23.53)	0.156	174.40 (150.00;203.52)	141.60 (114.60;194.32)	–27.40 (–37.20; –4.65)	**0.010**	0.349	0.737
SVR (dynes/cm^5^)	1290.80 (1138.60;1506.60)	1199.10 (994.87;1451.45)	–140.05 (–291.23;63.58)	0.126	1319.70 (1179.20;1510.80)	1151.80 (987.60;1285.00)	–71.50 (–334.55; 58.30)	**0.028**	0.873	0.584

Data are expressed as median (interquartile range). CR, cardiac rehabilitation; SSRV, stroke systemic vascular resistance; SSRVI, stroke systemic vascular resistance index; SVR, systemic vascular resistance.

*p*: values of comparison between changes of pre-treatment and post-treatment observed in CR group or control group.

*p: values of comparison between baseline parameter in CR group versus those in control group.

^#^*p*: values of comparison between parameter after treatment observed in CR group versus those in control group.

*p* < 0.05 was considered statistically significant.

In control group, the SVR, SSVR, and SSVRI showed no differences before and after the treatment. In CR group, the SSVR decreased from 300.70 (210.40; 352.07) dynes/cm^5^/beat to 251.45 (175.35; 318.85) dynes/cm^5^/beat (*p* < 0.05), SSVRI decreased from 174.40 (150.00;203.52) dynes/cm^5^/m^2^/beat to 141.60 (114.60;194.32) dynes/cm^5^/m^2^/beat (*p* < 0.05), and SVR decreased from 1,319.70 (1,179.20; 1,510.80) dynes/cm^5^ to 1,151.80 (987.60; 1,285.00) dynes/cm^5^ (*p* < 0.05). The results above suggested that phase 1 CR plus routine treatment could decrease system resistance to lower pressure load.

### The correlation between changes in impedance cardiography parameters and NT-proBNP

Spearman’s correlation analysis was used to identify the correlation between the changes in ICG parameters and NT-proBNP (Table 9); however, no correlation was observed between these two items.

**TABLE 9:** Correlation between change of hemodynamic parameters measured by impedance cardiology and change of NT-proBNP in patients with CHD and AHF.

	All group R	*P*	Control group R	*P*	CR group R	*P*
ΔCO	–0.3	0.895	0.101	0.709	–0.344	0.117
ΔCI	0.087	0.605	0.117	0.667	0.035	0.878
ΔSV	–0.019	0.912	0.069	0.799	–0.212	0.344
ΔSI	0.022	0.904	0.074	0.786	–0.093	0.742
ΔTFC	–0.148	0.376	–0.404	0.121	0.074	0.744
ΔSSVR	–0.074	0.693	–0.114	0.674	0.076	0.789
ΔSSVRI	–0.078	0.765	–0.122	0.654	0.112	0.690
ΔSVR	–0.127	0.446	–0.223	0.407	0.125	0.579
ΔPEP	0.053	0.777	0.073	0.789	–0.129	0.646
ΔLVET	0.040	0.831	0.022	0.936	0.207	0.460
ΔSTR	0.092	0.622	0.036	0.896	0.260	0.349

Δ = post-treatment–pre-treatment.

*p* < 0.05 was considered statistically significant.

## Discussion

Previous studies have demonstrated that hemodynamic parameters measured by ICG can reflect the hemodynamic characteristics of patients with HF. In this study, we found that both standard pharmacotherapy and phase 1 CR combined routine medical treatment could lower NT-proBNP levels in patients with CHD and AHF. However, the number and the percentages of high-risk patients in CR group significantly decreased after the treatment. Meanwhile, most hemodynamic parameters improved after the treatment in CR group, but not in control group, suggesting that phase 1 CR plus standard pharmacotherapy improved hemodynamic characteristics by elevating SV, CO, and CI, decreasing TFC in high-preload patients, shortening PEP and STR, and lowering SVR, SSVR, and SSVRI, although the post-treatment hemodynamic parameters showed no statistically significant differences between control group and CR group. Most importantly, the decrease of standardized percentage of patients with dysfunctional problem in CR group was higher than that in control group, indicating an improvement in systolic and diastolic function of left ventricular.

### NT-proBNP and cardiac rehabilitation

Large amounts of studies have demonstrated that the level of NT-proBNP is a predictor of outcome of patients with HF (24, 25). Normally, the higher level of NT-proBNP, the higher of New York Heart Association (NYHA) functional classes, the worse of prognosis of patients with HF (26). Meanwhile, in this study, both routine treatment and routine treatment combined phase 1 CR could decrease the level of NT-proBNP effectively in patients with CDH and AHF. Subgroup analysis demonstrated that the number and the percentages of patients with higher level of NT-proBNP in the CR group significantly decreased (*p* < 0.05), suggesting that phase 1 CR plus medication can further lower the levels of NT-proBNP in patients with CHD and AHF, which implies that phase 1 CR may improve the outcome in patients with HF. Previous studies have found that phase 1 CR could improve the symptoms of patients with HF. A perspective study showed that phase 1 CR improved the percentage of patients in NYHA class I and class II from 19.6 and 35.2% in the admission to 24.8 and 54.1% in the dismission, respectively, and the percentage of patients with NYHA class III decreased from 44.2 to 19.6% (6). Similarly, Taya et al. found that the high-intense intermittent training decreased the serum level of BNP in patients with HF from 432 (812) pg/ml to 254 (400) pg/ml (*p* < 0.001) (27). Those results imply that early movement can improve the symptoms of patients with HF. Moreover, Motoki et al. demonstrated that phase 1 CR improved the daily activity function in patients with acute decompensated HF (7).

### Impedance cardiography and cardiac rehabilitation

#### Cardiac function

Several reports have demonstrated the accuracy of SV and CO detected by ICG (28, 29). We found that patients in CR group had a significant improvement in SV, CO, and CI and a non-significant improvement in SI. However, no significant differences were observed after the treatment in all parameters above mentioned between control group and CR group. The improvement of post- and pre- treatment SV in the CR group, not in control group, implied that early movement may improve pump function of heart. Consistent with our finding, Chursina et al. reported that free-load bicycle exercise improved LVEF in patients with ischemic cardiomyopathy (30, 31).

#### Preload

Thoracic fluid content is used to reflect the preload or volume load in ICG. Previous studies have found that TFC is negatively correlated with pulmonary capillary wedge pressure (32). The patients with severer symptoms and higher NYHA levels had higher TFC level in AHF (33). Moreover, TFC in patients with HF had a significant positive correlation with re-admission rate and risk of death in 2 months (34). However, there is no research deciphering the effect of phase 1 CR on TFC. In this study, phase 1 CR plus routine medical treatment only showed a decrease tendency in TFC. Interestingly, in patients with TFC ≥ 0.035/Ω, phase 1 CR plus medical treatment significantly decreased TFC. However, such improvement was not discovered in patients with TFC < 0.035/Ω. Taken together, our findings suggested that phase 1 CR decreased preload of heart in patients with high preload. Consistently, Gielerak et al. found that 8-week CR could decrease TFC, elevate the maximum oxygen uptake, and improve exercise tolerance in patients with HF (35). Moreover, the decrease in TFC had positive correlation with the improvement in 6-min walk test (36). All the results above suggest that CR can improve cardiac function by decreasing TFC level.

#### Contraction

Pre-ejection period refers to the time period of isovolumic contraction and LVET refers to time period of left ventricular isometric contraction. The decreased contraction would result in prolonging PEP and shortening LVET. STR is the ratio of PEP and LVET. Thus, STR can reflect the efficiency of ventricular contraction and left ventricular function.

In this study, the significant decrease of PEP and STR in CR group, not in control group, implied that phase 1 CR combined medical treatment improved left ventricular contraction in patients with CHD and AHF, although no significant differences have been found between control group and CR group. Emerging studies have demonstrated the correlation of STR and risk of death in patients with HF. Sadauskas et al. reported that STR ≥ 0.55 is an indicator of higher risk to death in 6 months in patients with recurrent HF (OR = 0.29) (37). Moreover, in PREDICT trial, a multicenter trial including 2,316 patients, the hemodynamic parameters including STR were identified as a predictor of short-term clinical events, such as HF recurrent decompensation (21). The findings above suggest that STR could be used to warn the risk of adverse cardiovascular events.

Furthermore, STR is related to short-term outcome of patients with HF (21). Thompson reported the negative correlation between STR and LVEF (*r* = –0.54; *p* < 0.001) (38). Vijayaraghavan et al. analyzed ICG parameters and quality of life in 64 patients with chronic HF and pointed out that shorter PEP was associated with the improvement in NYHA level (39), suggesting that PEP could be a reflection of symptoms in patients with HF.

In addition, STR can imply diastole function. In IMPEDDANS study, ICG was used to evaluate diastolic dysfunction in patients with arterial hypertension. Nazario Leao et al. found that PEP, LVET, and STR had good discriminative ability in discovering left ventricular diastole dysfunction. Amid them, the sensibility of STR was 99% and the specificity was 90%. The threshold of diastole dysfunction is PEP ≤ 104 ms, LVET ≥ 320 ms, and STR ≤ 0.31 (40). Although we did not focus on diastolic improvement, the results of our study implicated that phase 1 CR combined medical treatment could shorten PEP and STR, which suggests the improvement of phase 1 CR on diastole function in patients with CHD and AHF worthy further investigation.

#### Afterload

Systemic vascular resistance, SSVR, and SSVRI are the parameters that reflect pressure load in ICG. Cotter et al. found an elevation in SVRI in patients with AHF (41), which was due to the activation of neuroendocrine. The elevation of SVRI contributed to the persistence of blood pressure and perfusion of important organs under the circumstance of decreased contractility. Overload SVRI may cause elevated afterload, decreased CI, increased left ventricular end-diastolic pressure and pulmonary capillary wedge pressure, and eventually leading to pulmonary edema. While decreasing SVR properly can increase SV, which could improve pulmonary congestion, however, there is no research indicating the effects of phase 1 CR on cardiac afterload. In this research, the significant decrease of SVR, SSVR, and SSVRI in CR group, but not in control group, suggested that phase 1 CR combined medical treatment could reduce afterload, at least to some extent, in patients with CHD and AHF, although no significant differences of post-treatment SVR, SSVR, and SSVRI have been found between control group and CR group.

### Correlation of BNP and impedance cardiography

Previous studies identified some correlations between BNP and ICG parameters. As Pomenta et al. reported, the TFC measured by ICG is an independent predictor of BNP in patients with AHF, despite the severe contraction dysfunction and NYHA levels (34). However, in this study, we did not determine the correlation between NT-proBNP and ICG parameters.

### Long-term outcomes

Despite that 1-week period of phase 1 CR improved cardiac function and hemodynamic characteristics in patients with CHD and AHF, whether the short-term benefits remain in the long run still needs to be further studied. Of note, though emerging studies have suggested that long-term exercise can improve the outcomes in patients with HF, the participation of long-term exercise is relatively low. Whether patients completed phase 1 CR would choose to continue phase 2 and phase 3 CR depends on the willing of patients, the recommendation of physicians, and the convenience, that is, whether there are facilities near the their communities (5). Thus, facilities need to be built and physicians should take the responsibility to recommend patients to further exercise movements.

## Limitations

This study has several limitations. Frist, the sample amount was relatively small due to the limitation of collection time and one-center study. Second, this study did not compare the effects of phase 1 CR in population with HF with preserved EF, HF with might reversed EF, and HF with reserved EF. Therefore, more studies with larger population and multicenters were needed to confirm the effects of phase 1 CR on cardiac function and hemodynamics in patients with CHD and AHF.

## Conclusion

In this study, we find that phase 1 CR plus routine medication can improve cardiac function and hemodynamic parameters in patients with CHD and AHF in short term. Thus, it is important to recommend phase 1 CR to patients once they are stable.

## Data availability statement

The raw data supporting the conclusions of this article will be made available by the authors, without undue reservation.

## Ethics statement

The studies involving human participants were reviewed and approved by the Clinical Research Ethics Committee, the Second Xiangya Hospital of Central South University. The patients/participants provided their written informed consent to participate in this study.

## Author contributions

All authors listed have made a substantial, direct, and intellectual contribution to the work, and approved it for publication.
